# Molecular Modeling and MM-PBSA Free Energy Analysis of Endo-1,4-β-Xylanase from *Ruminococcus albus* 8

**DOI:** 10.3390/ijms151017284

**Published:** 2014-09-26

**Authors:** Dongling Zhan, Lei Yu, Hanyong Jin, Shanshan Guan, Weiwei Han

**Affiliations:** 1 Key Laboratory for Molecular Enzymology and Engineering of Ministry of Education, Jilin University, Changchun 130023, China; E-Mails: zdlgale@126.com (D.Z.); gykfamily@163.com (L.Y.); hyjin13@mails.jlu.edu.cn (H.J.); 2 College of Food Science and Engineering, Jilin Agricultural University, Changchun 130118, China; 3 State Key Laboratory of Theoretical and Computational Chemistry, Institute of Theoretical Chemistry, Jilin University, Changchun 130023, China; E-Mail: guanss12@mails.jlu.edu.cn

**Keywords:** homology modeling, molecular dynamics, MM-PBSA, endo-1,4-β-xylanase

## Abstract

Endo-1,4-β-xylanase (EC 3.2.1.8) is the enzyme from *Ruminococcus albus* 8 (*R. albus* 8) (Xyn10A), and catalyzes the degradation of arabinoxylan, which is a major cell wall non-starch polysaccharide of cereals. The crystallographic structure of Xyn10A is still unknown. For this reason, we report a computer-assisted homology study conducted to build its three-dimensional structure based on the known sequence of amino acids of this enzyme. In this study, the best similarity was found with the *Clostridium thermocellum* (*C. thermocellum*) *N*-terminal endo-1,4-β-d-xylanase 10 b. Following the 100 ns molecular dynamics (MD) simulation, a reliable model was obtained for further studies. Molecular Mechanics/Poisson-Boltzmann Surface Area (MM-PBSA) methods were used for the substrate xylotetraose having the reactive sugar, which was bound in the −1 subsite of Xyn10A in the ^4^*C*_1_ (chair) and ^2^*S_O_* (skew boat) ground state conformations. According to the simulations and free energy analysis, Xyn10A binds the substrate with the −1 sugar in the ^2^*S_O_* conformation 39.27 kcal·mol^−1^ tighter than the substrate with the sugar in the ^4^*C*_1_ conformation. According to the Xyn10A-^2^*S_O_* Xylotetraose (X4(sb) interaction energies, the most important subsite for the substrate binding is subsite −1. The results of this study indicate that the substrate is bound in a skew boat conformation with Xyn10A and the −1 sugar subsite proceeds from the ^4^*C*_1_ conformation through ^2^*S_O_* to the transition state. MM-PBSA free energy analysis indicates that Asn187 and Trp344 in subsite −1 may an important residue for substrate binding. Our findings provide fundamental knowledge that may contribute to further enhancement of enzyme performance through molecular engineering.

## 1. Introduction

*Ruminococcus albus* 8 (*R. albus* 8) is one of the most actively fibrolytic ruminal bacteria in the world, which can degrade cellulose and hemicellulose in forages such as alfalfa and grass hays [1,2,3]. It is well known that *R. albus* 8 has a wide range of protein activities [4,5,6].

Xylans are abundant hemicellulolytic components of the plant cell wall, which can be degraded into the corresponding oligomeric and monomeric sugars, providing a major source of renewable energy. The main chain of xylan is composed of 1,4-d-xylose subunits, which is usually decorated with various side chain residues of 1,2-α-d-glucuronic acid, or its 4-*O*-methyl ethers, 1,3-α-l-arabinose, and/or *O*-acetyl groups in the 2 and 3 positions. Due to structural complexity, several xylanolytic enzymes are required to release the substituents and sugars from the various xylans, including endo-1,4-β-xylanases (EC 3.2.1.8) [7], acetyl xylan esterases (EC 3.1.1.72) [8], feruloyl esterases (EC 3.1.1.73) [9], α-l-arabinofuranosidases (EC 3.2.1.55) [10], α-glucuronidases (EC 3.2.1.139) [11], and β-d-xylosidase (EC 3.2.1.37) [12]. Among these enzymes, endo-1,4-β-xylanases are believed to be the most valuable in industrial applications.

Endo-1,4-β-xylanase (EC 3.2.1.8) is the enzyme from *R. albus* 8 that catalyzes the degradation of arabinoxylan, a major cell wall non-starch polysaccharide of cereals [13]. It is well known that cellulases and xylanases are key enzymes involved in the degradation of β-glucan biomass, acting through hydrolysis of the β-glycosidic linkages. All members of a family exhibit the same catalytic mechanism: the stereochemistry at the anomeric center of the bond being cleaved is either inverted or retained as a consequence of hydrolysis. Inverting glycosidases appear to use a mechanism in which a general acid/base catalyzed direct displacement occurs at the anomeric center through an oxocarbenium ion-like transition state [14,15]. Retaining glycosidases, however, use a double-displacement mechanism in which a covalent glycosyl-enzyme intermediate is formed and hydrolyzed in a general acid/base catalyzed process through oxocarbenium ion-like transition states, or possibly through oxocarbenium ion intermediates [16,17].

Most of the xylanases are classified in glycoside hydrolase families (GH)10 and GH11, having distinct structures [18,19]. Phytopathogenic microorganisms use GH10 and GH11 xylanases to infect plants [20,21,22,23]. Many xylanases are used in breadmaking [24,25,26], animal feeding [27], and gluten–starch separation [28,29].

GH10 xylanases have a (β/α)_8_-barrel as a catalytic domain and typically contain one or more carbohydrate-binding domains, which increase the effective concentration of the active site on polymeric substrates [29]. The structure of GH11 xylanases has been described as a partially closed right hand. In contrast with GH10 xylanases, no carbohydrate-binding modules are present in GH11 [29]. A consequence of the difference in structure can lead to the difference in substrate specificity. It was reported that GH10 xylanases have a lower number of unsubstituted consecutive xylose units [29]. In contrast, GH11 xylanases show higher affinity towards a larger number of unsubstituted consecutive xylose units because of their larger active site.

The reaction mechanism of both GH10 and 11 xylanases is a general acid–base mechanism resulting in retention of the anomeric configuration in the product. The mechanism is a double displacement in which a covalent intermediate is formed and then hydrolyzed via oxocarbenium ion-like transition states [30,31]. It involves two acidic amino acid residues, one acting as an acid/base and one as a nucleophile. For xylanases of GH10 and 11, these two catalytic residues are glutamate residues.

However, the endo-β-1,4-xylanase in *R. albus* 8 (Xyn10A) has received limited investigation. Previous studies on *R. albus* 8 have focused on cellulose-degrading enzymes (Cel5G, Cel9B, Cel9C, and Cel48A) and rarely on its hemicellulases [13]. Until now, the 3D structure of Xyn10A was not known. With the development of computer technology, it is possible for us to build the 3D structure of the protein through homology modeling. In this study, the 3D structure of Xyn10A was built and used to predict the binding pose between the enzyme and ligands.

## 2. Results and Discussion

### 2.1. Relatedness of the GH10 Family

**Figure 1:**
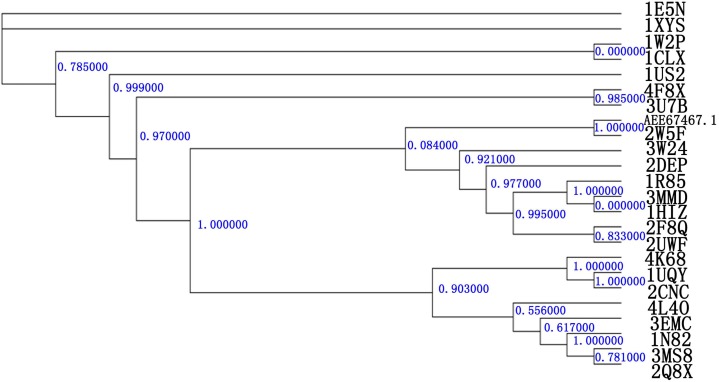
A phylogenetic tree of GH10 family. R. albus 8 (Xyn10A) (No. AEE64767.1). The known 3D structures of the GH10 family were downloaded from the CAZY database [35]. The tree was calculated using the PhyML program [36] with the LG (Si Quang Le and Olivier Gascuel) substitution matrix γ distribution. Xyn10A and Xyn10b were made up one clade.

A phylogetic analysis of Xyn10A with various GH10 families is shown in Figure 1. Xyn10A clusters together with *C. thermocellum* *N*-terminal endo-1,4-β-d-xylanase 10 b (Xyn10b) (PDB Id 2W5F) [19]. Thus, this suggests that Xyn10A and *C. thermocellum* *N*-terminal endo-1,4-β-d-xylanase 10 b (Xyn10b) will form a new clade in GH10 family. When analyzed at the derived protein level, there was about 39% identity for Xyn10A and Xyn10b. In the GH10 family parsimony tree, Xyn10A and Xyn10b formed a separate but related clade, and shared about 40% amino acid sequence identity with various GH10 xylanases. It is suggested Xyn10A may have evolved from a common β-glycosidase ancestor and share a similar binding site.

### 2.2. Homology Modeling

Several three-dimensional structures with homologous sequences to Xyn10A were found with PDB/BLAST. Seven templates were used to build the model (Table 1). The 3D structure of Xyn10A was built by Swiss model in line with the latest version [32]. The improved Swiss model pipeline makes extensive use of model quality estimation for selection of the most suitable templates and provides estimates of the expected accuracy of the resulting models [32].

The estimates of the expected accuracy of the resulting models were listed in Table 1. From Table 1, 99.3% residues of the model made of Xyn10b (PDB Id 2W5F) were in the allowed region by PROCHECK methods [33]. The Verify_3D score [34] of the model made of Xyn10b (PDB Id 2W5F) (86.09% residues > 0.2) was higher than the other seven models. It is well known that homology modeling relies on evolutionarily related structures (templates) to generate a structural model of a protein of interest (target) [32]. Xyn10A and *C. thermocellum* *N*-terminal endo-1,4-β-d-xylanase 10 b (Xyn10b) will form a new clade in the GH10 family, and thus they may share similar protein folding and active pockets. At same time, the resolution of the template PDB Id 2W5F A is 1.90 Å, indicating that it can be seen as a reliable template. Global QMEAN estimates are provided as a Z-score that relates the obtained values to scores calculated from a set of high-resolution X-ray structures [32]. The resulting GMQE (Global Model Quality Estimate) is expressed as a number between zero and one, where higher numbers indicate higher reliability. In Table 1, the global model quality estimate between the model and the template (PDB Id 2W5F A) is 0.66, which indicates that the model made with Xyn10b (PDB Id 2W5F A) has fair accuracy.

**Figure 2:**
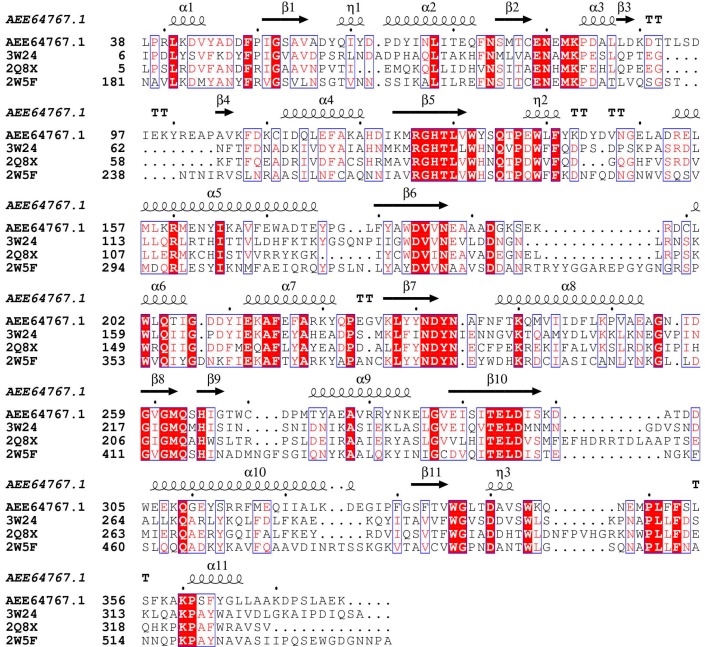
Sequence alignment of Xyn10A with other members in the GH10 superfamily. Xyn10A: (GenBank: AEE64767.1) [13]; 3W24: intracellular xylanase from Thermoanaerobacterium saccharolyticum JW/SL-YS485(PDB Id 3W24) [7]; 2Q8X: the thermophilic, intracellular xylanase from G. Stearothermophilus (PDB Id 2Q8X) [38]; 2W5F: the *N*-terminal endo-1,4-β-d-xylanase 10 b (Xyn10b) of C. thermocellum (PDB Id 2W5F) [19]. Strictly conserved residues are highlighted by red background and conservatively substituted residues are boxed. The secondary structural elements (helices-α, strands-β, turns-T) of Xyn10A are shown above the aligned sequences. The conserved catalytic residues E186 and E294 are indicated by black triangle. The figure was produced using ESPript [39].

Four proteins sequences were obtained from the NCBI database and aligned as shown in Figure 2. At the primary structure level, the alignment of the amino acid sequences of Xyn10A and the Xyn10b showed a high degree of similarity, especially in the catalytic site, as can clearly be observed (in Xyn10A Glu188 acts as the catalytic acid/base, and Glu294 acted as catalytic nucleophile) in Figure 2. A high percentage of similarity was found (above 39%), which was adequate enough to build a reliable model. For the reason discussed above, the 3D structure of Xyn10b (PDB Id 2W5F A) was chosen as a template. In this paper, the model was made up of the residues from 36 to 372.

**Table 1:** The estimates of the expected accuracy of the resulting models results.

Template (PDB Id)	Sequence Identity	Resolution	Organism	Global Model Quality Estimate	Procheck	Verify_3D
2W5F A	39%	1.90	*C. Thermocellum*	0.66	86.6% core 11.8% allow 1.0% gener 0.7% disall	86.09% residues > 0.2
2WZE A	39%	2.50	*C. Thermocellum*	0.63	82.3% core 14.7% allow 2.0% gener 1.0% disall	83.33%
3W24 A	38%	1.32	*Thermoanaerobacterium saccharolyticum*	0.63	87.0% core 11.0% allow 1.7% gener 0.3% disall	82.53%
2Q8X A	38%	1.45	*G. stearothermophilus*	0.61	84.2% core 13.4% allow 1.0% gener 1.3% disall	73.94%
3MS8 A	39%	1.70	*G. stearothermophilus*	0.60	81.2% core 18.1% allow 0.3% gener 0.3% disall	69.70%
3MUI A	39%	1.80	*G. stearothermophilus*	0.61	83.6% core 14.4% allow 1.7% gener 0.3% disall	70.30%
1N82 A	38%	1.45	*G. stearothermophilus*	0.60	81.7% core 16.6% allow 0.3% gener 1.3% disall	83.70%

**Figure 3:**
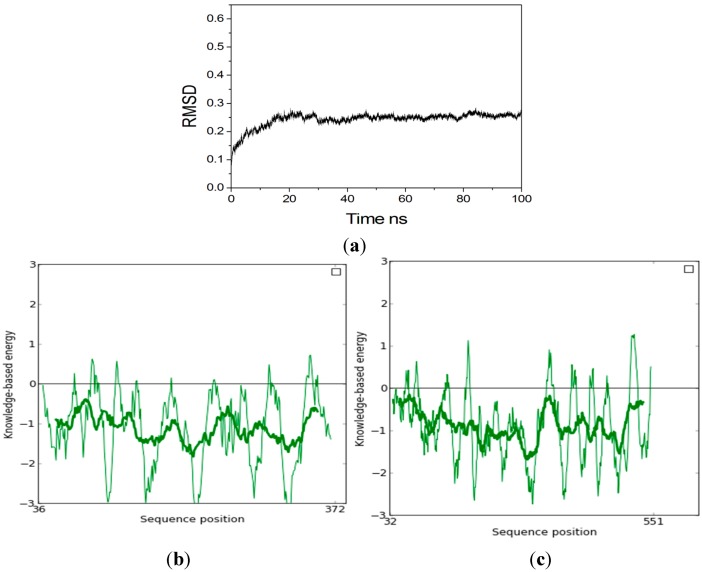
(**a**) The *C*α RMSD during the 100 ns MD. Screen shot of residue of a native protein (**b**) Xyn10A; (**c**) Xyn10b. Thin line represents result of window size 10, thick line represents result of window size 40.

**Figure 4:**
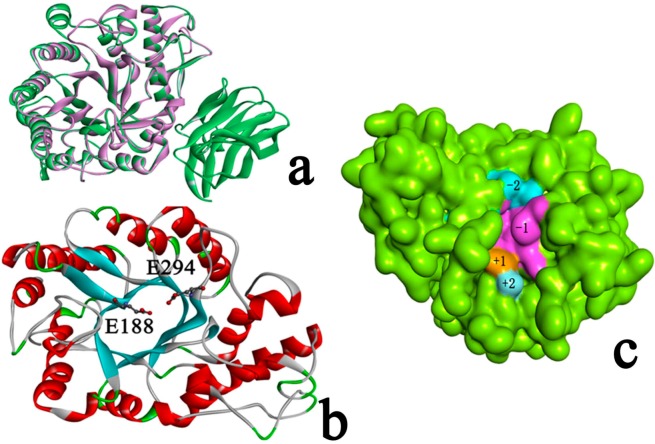
(**a**) Superimposition of Xyn10A (purple) and the template Xyn10b (green) (RMSD 0.32 Å); (**b**) The 3D structure of Xyn10A. Glu188 and Glu294 located at the end of β-strands 4 and 7, repetitively; (**c**) The surface of Xyn10A. Color light blue represents for subsite +2, color brown represents for subsite +1, color purple represents for subsite −1, and color dark blue represents for subsite −2.

The model was minimized using the Amber 11.0 program with the conditions described. Figure 3a shows the RMSD of *C*α atoms with respect to their initial positions during 100 ns MD simulations. The protein was stable observed from the plot during the first 30,000 ps. So the last conformation during the 100 ns MD simulation was chosen for further study. The stereochemistry of the model was assessed using ProSA-web [37], which is a diagnostic tool that is based on the statistical analysis of all available protein structures. The location of the *Z*-score for Xyn10b (PDB Id 2W5F A is −10.49, and is in the range of native conformations, and the location of the *Z*-score for Xyn10A is −9.03 and is also in the range of native conformation similar to Xyn10b. Figure 3b,c show the screen shot of residue of a native protein, indicating that the two structures of Xyn10A and Xyn10b are similar to each other. Figure 4a shows the superimposed alignment of Xyn10A after 100 ns MD simulations (pink) and the template, Xyn10b (PDB Id 2W5F) (green). The RMSD of *C*α atoms was 0.32 Å, which indicated that the two structures were very similar. Table 2 listed that the estimates of the expected accuracy of the initial model the last conformation during 100 ns MD. From the two different checks, the structure of Xyn10A after 100 ns MD is more accurate than the initial model’s, and thus it can be used for further study.

**Table 2:** The estimates of the expected accuracy of the initial model of the last conformation during 100 ns MD.

Protein	Procheck	Verify_3D
The initial model	86.6% core 11.8% allow 1.0% gener 0.7% disall	86.09% residues > 0.2
The last conformation model	88.5% core 10.0% allow 1.1% gener 0.4% disall	97.66%

Figure 4b shows the 3D structure of Xyn10A. Xyn10A has a typical (β/α)_8_ barrel fold. The family GH10 endo-1,4-β-xylanase catalyses the hydrolysis of xylan by means of a double displacement mechanism via oxocarbenium-like transition states, with net retention of configuration [31,40].

### 2.3. Identification of Binding Site in Xyn10A

Subsite mapping of glycosyl hydrolases began in the late 1960s [41]. The xylanases’ subsites towards the reducing end of the substrate are labelled −1, −2, −3 to −*n* and those towards the non-reducing end, away from the point of cleavage, 1, 2, 3 to *n* [41]. Consistent with their endo-mode of action GH10 xylanases contain an extended substrate-binding cleft that can accommodate between four to seven xylose residues [19]. Each region that binds to a xylose moiety is known as a subsite, which can be given negative or positive numbers depending on whether they recognize sugars that are upstream or downstream of the active site (named subsite −2, −1, +1, and +2 see form Figure 4c) [19]. Unlike the template, Xyn10A has only GH10 domain, and has no CBM22 (CBM22-2), a dockerin sequence and a *C*-terminal family 1 carbohydrate esterase (CE1) catalytic domain. It was reported that residues from helix H4 of the GH10 module provide the major contacts by fitting into the minor groove of the CBM22-1 module. And thence the orientation of CBM22-1 would allow the substrate to be loosely bound and subsequently delivered to the active site in a possessive manner. The difference between the substructure of Xyn10A and the template may lead to catalytic efficiency of the two proteins.

Xyn10A mediates hydrolysis of the glycosydic bond via a general acid/base double displacement mechanism that results in retention of anomeric configuration. Catalysis requires a catalytic acid/base and a catalytic nucleophile. In Xyn10A, Glu188 and the catalytic nucleophile, Glu294, are located at the end of β-strands 4 and 7 (equivalent to β-strands 4 and 7 in Xyn10b), respectively (Figure 4c).

In family GH10 enzymes, hydrolysis often occurs between the −1 and +1 subsites, and thus the subsite −1 essentially comprises the active site. According to the sequence to the templates Xyn10b (PDB Id 2W5F) [19] and xylanase from Thermoanaerobacterium saccharolyticum (PDB Id 3W24) [7], in subsite −1 for Xyn10A, there are seven residues: Gln263, Lys83, W336, Glu188, Glu294, His128, Met87, His265, and Trp344. All these residues are conservative in family GH10 seen from Table 3. For the subsite −2, there are Glu79, Asn80, and Trp132 in subsite −2. For the subsite +1, there is Tyr233 in the subsite +1. For the subsite +2, there is Asn234 in the subsite +2. These residues are also conserved, meaning that these residues are also important for enzyme catalysis and substrate binding.

**Table 3:** The subsite −1 residues in GH10 family.

Protein/Residue	263	265	128	187	344	336	83
Xyn10A	Q	H	H	N	W	W	K
2W5F	Q	H	H	N	W	W	K
3W24	Q	H	H	N	W	W	K
2Q8X	Q	H	H	N	W	W	K

### 2.4. Docking Study

#### 2.4.1. Structures of Ground State Complexes

**Figure 5:**
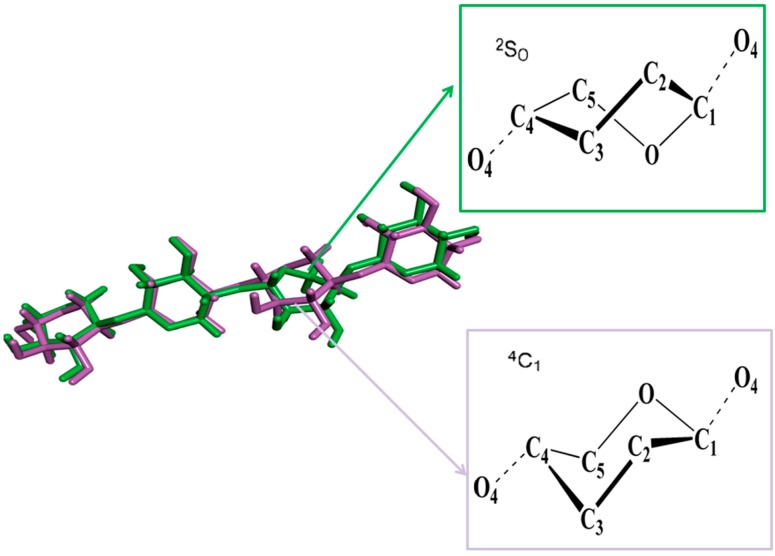
Two xylotetraose conformations were selected for further simulations: one with the −1 sugar in the 2SO conformation (marked as X4(sb) and the rest in the 4C1 conformation, and another with all sugars in the 4C1 conformation (marked as X4(c).

The endo-1,4-β-xylanase from *Trichoderma reesei* belongs to family 11 of the xylanases and it has an active site geometry in which the proton donor interacts with the scissile glycosidic bond from the side of the carbohydrate plane allowing synprotonation [40]. Xylotetraose (X4) was docked to subsites −2, −1, +1, and +2 using the complex structure of Bacillus cir*culans* xylanase mutant E172C as a reference [42], whereas several orientations were considered for xylose residues occupying subsites −1. Two xylotetraose conformations were selected for further simulations: one with the −1 sugar in the 2SO conformation (marked as X4(sb) and the rest in the 4C1 conformation, and another with all sugars in the 4C1 conformation (marked as X4(c) (see Figure 5). These structures had no significant steric overlap and they fulfilled the geometrical requirements for the lateral protonation of the scissile glycosidic bond in the enzymatic reaction [40].

**Figure 6:**
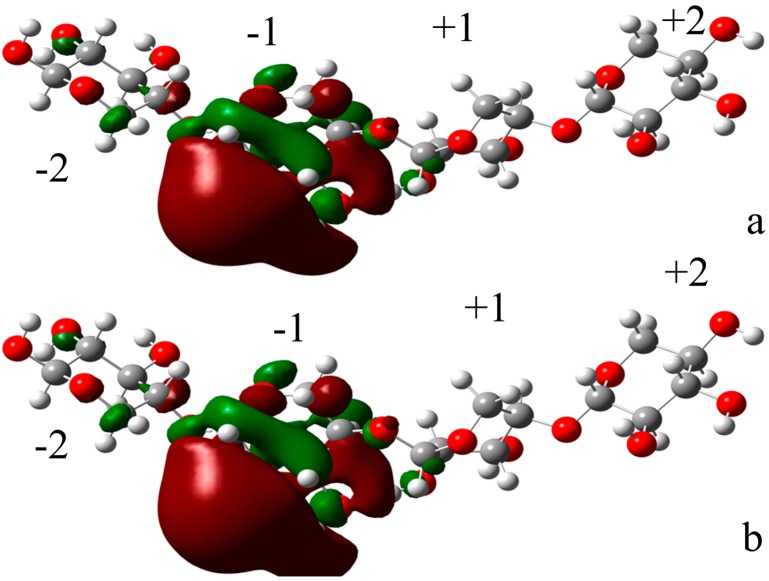
(**a**) The LUMO orbit of X4(sb); (**b**) The LUMO orbit of X4(c).

Two xylotetraose conformations were optimized with B3LYP/6-31G * set with Gaussian 03 software [43].Seen from Figure 2, the HOMO orbit and the LUMO orbit of xylotetraose indicated that the phenol ring is the active center of xylotetraose. The energy between the HOMO and he LUMO (Egap) is 175.5 kcal·mol^−1^ for X4(sb) and Egap is 182.57 kcal·mol^−1^ for X4(c). The less energy for X4(sb) indicated that is easy to bind to the enzyme. As shown in Figure 6a,b, the LUMO orbit of X4(sb) and X4(c) indicates that the subsite −1 is the active center of the two substrate.

#### 2.4.2. Docking Validation

Despite many challenges, docking methods have emerged to become useful tools in drug discovery and design [44]. In particular, it is important to ascertain how well a given procedure can accurately generate and score known ligand binding poses [45].

**Figure 7:**
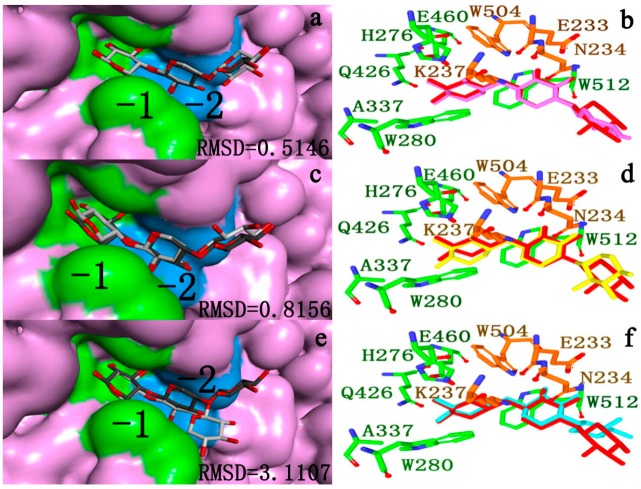
(**a**) the compartment between the docked ligand (dark gray) and the reference for the crystal structure (light gray) located in the active cleft. Calculated by CDOCKER [46]; (**b**) the subsite −1 and −2 residues around the docked ligand (purple) and the reference for the crystal structure (red); (**c**) the compartment between the docked ligand (dark gray) and the reference for the crystal structure (light gray) located in the active cleft. Calculated by Autodock vina [47]; (**d**) the subsite −1 and −2 residues around the docked ligand (yellow) and the reference for the crystal structure (red); (**e**) the compartment between the docked ligand (dark gray) and the reference for the crystal structure (light gray) located in the active cleft. Calculated by Autodock 4.2 [48]; (**f**) the subsite −2 and −1 residues around the docked ligand (dark blue) and the reference for the crystal structure (red).

Docking success was observed when the top scoring pose was about 2.0 Å heavy atoms RMSD of the crystal ligand [44]. It is important to note that examining docking accuracy depends on the RMSD algorithm employed. When the top-scoring pose was not within 2.0 Å, it was defined as a scoring failure. Figure 7a–f shows a representative example for a ligand docked to a target (PDB Id 2W5F) with CDOCKER [46], Autodock vina [47] and Autodock 4.2 [48] software. Seen from Figure 7a–f, the docked ligands were in the same orientation in the different binding modes (−2, −1, +1, +2). And it is easier to see which part of the ligands docks in which cavity. In comparison to the crystallographic reference, the ligand docked by CDOCKER was successful (RMSD0.5416). So CDOCKER was used for further docking analysis.

**Figure 8:**
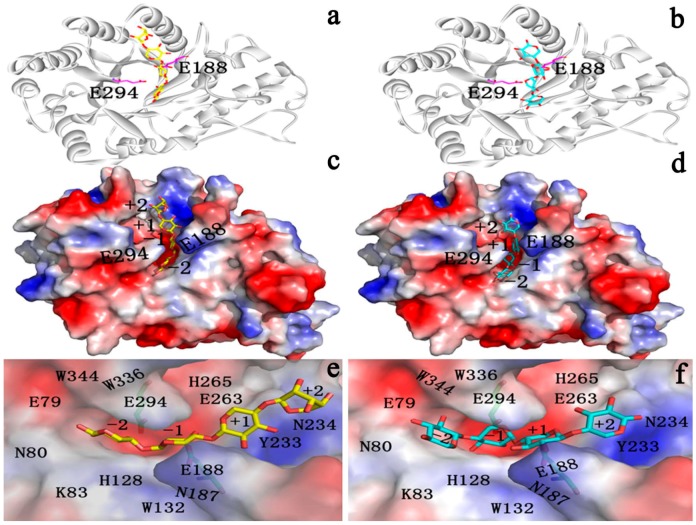
The docked complex. All α-helices and β-sheets are colored in white, and two catalytic glutamates (E188 and E294) are shown as sticks. (**a**) X4(sb)–Xyn10A complex; (**b**) X4(c)–Xyn10A complex (**c**) Surface representation and xylotetraose-binding mode of (**c**) X4(sb) in Xyn10A (**d**) X4(c) in Xyn10A. The protein is shown using the molecular electrostatic surface, with red for negative charge and blue for positive charge. Residues involved in the substrate-binding tunnel are labeled; (**e**) X4(sb) in Xyn10A; (**f**) X4(c) in Xyn10A.

The two substrates are docked in Xyn10A (Figure 8a–f). The xylose residues are clearly seen in the enzyme-substrate structure (Figure 8e,f). The two substrates are located in the active cleft. To study the properties of the reaction transition (intermediate) state, docking methods were utilized. The simulated structure represents a state where the glycosidic bond is cleaved, the glycosidic oxygen is protonated, the C1 carbon carries a positive charge and both catalytic glutamates are charged. This structure is thought to be close to the transition state of the retaining glycosyl hydrolases and therefore likely shows interactions important for transition state stabilization [49]. In the docked structure the glycosidic bond between the xylobioside parts of tetraose is cleaved and the distance between the C1 of the oxocarbenium ion and the glycosidic oxygen was set at 4.0 Å. With this restraint the newly formed OH is still in the close vicinity of the acid/base catalyst but not too close to the positively charged C1.

#### 2.4.3. Energy Analyses of the Complexes

**Figure 9:**
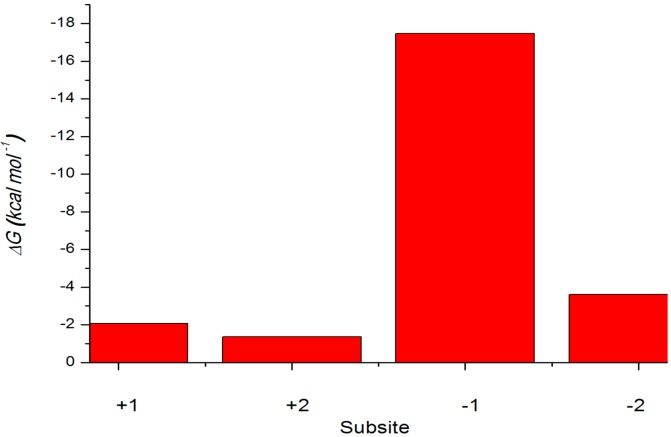
Internal molecular mechanical energies of xylose monomers in the enzyme. Energies of X4(sb) are shown with red (enzyme) columns.

The binding free energies for the two xylotetraose conformations X4(c) and X4(sb) were estimated (Table 4) using the MM-PBSA method. The calculated binding free energies were −1.67 kcal·mol^−1^ for X4(c), −40.94 for X4(sb) a. The analysis of the free energy components shows that the −47.09 kcal·mol^−1^ more favorable electrostatic interaction in X4(sb)–Xyn10A complex is the main reason for the higher affinity of X4(sb) as compared to X4(c). On the other hand, the van der Waals interaction is more favorable for X4(sb) conformation by 35.88 kcal·mol^−1^. The molecular mechanical energies of individual pyranoside subunits were examined using a strategy in which the substrate coordinates were taken from the molecular dynamics trajectories, the extracted structures were rebuilt as xylose monomers and the average internal molecular mechanical energies of the monomers and their interaction energies with the protein were calculated (Figure 9).

**Table 4:** The MM-PBSA score for the two complexes (kcal·mol^−1^).

Energy Components	X4(c)–Xyn10A	X4(sb)–Xyn10A
∆*E*_ele_	−40.72	−98.71
∆*E*_vdW_	−11.66	−47.24
∆*G*_PB_	53.63	112.02
∆*G*_np_	−2.92	−7.01
Nonpolar	−14.58	−54.25
Polar	12.91	13.31
∆*G*_bind_	−1.67	−40.94

The interaction energy of the reactive pyranoside unit in the subsite −1 is −17.47 kcal·mol^−1^ for the binding of the skew boat (Figure 9). In the case the protein–monomer interaction energies are within −1.37, −2.08 and −3.61 kcal·mol^−1^ in the subsites +2, +1 and −2. The subsite −2 is the least tightly bound of the four binding subsites with −3.61 kcal·mol^−1^ lower interaction energies than in the +2 and +1 subsites. According to the Xyn10A−X4(sb) interaction energies, the most important subsite for the substrate binding is subsite −1. The subsites −2 and −1 are also important for the tight binding and presumably for the correct positioning of the reactive sugar unit. That the sugar moiety of the −2 subsite is bound the least tightly of the four moieties may be due to the product release step of the reaction cycle: when the glycosidic bond is cleaved, the sugar moieties of the −1 and −2 subsites need to leave the substrate-binding site fast enough in order not to hinder the binding of the substrate of the next reaction.

#### 2.4.4. Computational Mutagenesis of Active Site Residues

**Figure 10:**
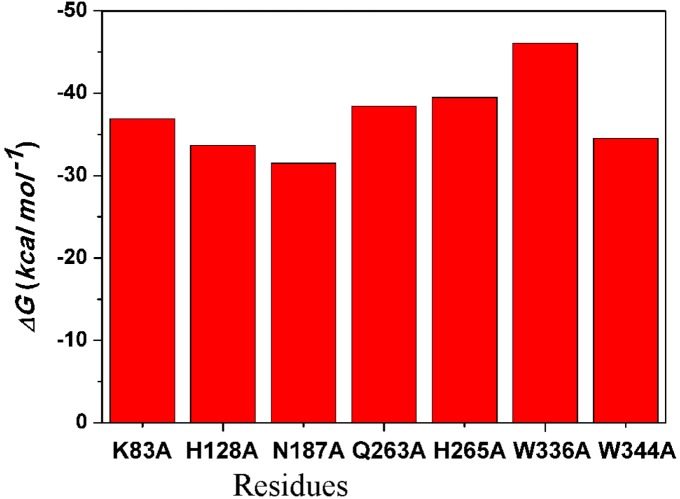
Computational alanine mutants (kcal·mol^−1^) calculated for the X4(sb)–Xyn10A (red columns).

Computational alanine scanning of selected amino acid residues of the active site groove was carried out to elucidate the role of individual residues on the binding of the substrate with the −1 sugar in the ^2^*S_O_* conformations. The residues mutated results of the mutagenesis are presented in Figure 10. It must be emphasized that the results of the computational mutagenesis of this study, one with a single trajectory method, are not meant to reproduce the corresponding energies measured experimentally, but to provide an estimate of the interactions between the sugar and the residue mutated in the original Xyn10A–X4(sb) complex. The W336A mutation lowers the affinity of X4(sb) by 5.14 kcal·mol^−1^. Other main components of the binding are residues K83, Q263, and H265, which all have little effects when mutated to alanine. The mutation N187A increases the affinity of X4(sb) by 8.79 kcal·mol^−1^. In addition, the mutation W344A increases the affinity of X4(sb) by 6.40 kcal·mol^−1^. And thence, Asn187 and Trp344 may an important residue for substrate binding.

## 3. Experimental Section

### 3.1. Homology Protein Modeling

To perform homology modeling for parts of the structure conserved among *XynA* with known crystal structures, previous target-template sequence alignment was performed using the Blast algorithm which gave the highest sequence similarity, 39%, to the *C. thermocellum* *N*-terminal endo-1,4-β-d-xylanase 10b (Xyn10b) CBM22-1-GH10 sequences [19]. Homology models using the comparative modeling module in Swiss model online produced reasonably good models [46]. Five steps are used in the protein modeling: (I) sequence alignment for generation of alignment based on one or more template structures; (II) threading for generation of initial models based on template structure by copying coordinates over the aligned regions; (III) loop modeling for rebuilding the missing parts using *de novo* modeling; (IV) selection of models based on reported experimental data from biochemical, biophysical, and electrophysiological studies; and (V) refinement using all-atom molecular dynamics (MD) simulations with reported constraints for the inter atomic distances of the salt-bridge interaction pair obtained from electrophysiology and mutagenesis experiments.

### 3.2. Molecular Dynamics (MD) Simulation

The model was then optimized using Amber 11 [50] for 100 MD simulation. The simulation was done in a truncated octahedral box under periodic boundary conditions and then neutralized with Cl^−^ counterions where necessary. Amber99sb force field was used for the protein–ligand complex. Prior to MD simulations, systems were energy minimized through the steepest descent algorithm with 2000 steps to avoid any steric conflicts generated during the initial setup. The density of the system was adjusted during the first equilibration runs at NPT condition by weak coupling to a bath of constant pressure (P_0_ = 1 bar, coupling time = 2 ps). For temperature regulation, we used Langevin thermostat (NTT = 3) to maintain the temperature of our system at 300 K. This temperature control method uses Langevin dynamics with a collision frequency of 1.0 ps [51]. As such, especially with explicit solvent dynamics, it is often better to equilibrate the system using Langevin methods (NTT = 3) [52] and then, once equilibrated, switch to Berendsen methods (NTT = 1) [53]. The electrostatic interactions were calculated by using the Particle-mesh Ewald (PME) algorithm [38]. The equilibration procedure consisted of thermalization of the solvent, for 500 ps at 300 K, followed by minimization of all solute atoms, keeping the solvent coordinates fixed, and then start of the MD simulation of the complete system by raising the temperature from 0 to 300 K in 500 ps increments of 50 K each. Data production was carried out for 10 ns for the two protein–ligands complex and 10 ns for the protein 10 under normal temperature (300 K) and pressure (1 bar), using a temperature coupling time constant of 0.1 ps and a pressure coupling time constant of 2.0 ps. The value of the isothermal compressibility was set to 4.5 × 10^−5^ bar for water simulations.

### 3.3. Docking Study

In validation analysis, AutoDock 4.2 [48], AutoDock vina [47], and CDOCKER [46] were used to perform docking analysis.

AutoDock 4.2 combines a rapid energy evaluation through precalculated grids of affinity potentials with a variety of search algorithms to find the best fit binding positions for a ligand to a given protein [48]. All torsion angles for each compound were considered flexible. The grid maps representing the proteins in the actual docking process were calculated with AutoGrid. The grids (one for each atom type in the ligand plus one for electrostatic interactions) chosen were sufficiently large enough to include not only active site but also significant portions of the surrounding surface [48].

AutoDock Vina is a new open-source program for drug discovery, molecular docking and virtual screening, offering multi-core capability, high performance and enhanced accuracy and ease of use [47].

CDOCKER [46] in the Discovery Studio 2.5 software package were used for their molecular docking into Xyn10A. During the two docking methods, the CHARMM force field was used [45].

### 3.4. Molecular Mechanics-Poisson–Boltzmann Surface Area (MM-PBSA) Analysis

The eight structures of the complex (the WT and the mutant with the substrate) were used as a starting point for calculating binding free energies. All the simulations were carried out by Amber 11 package for 10 ns using the amber99sb field force parameter [10]. The binding free energies were calculated by using the molecular mechanics-Poisson–Boltzmann surface area (MM–PBSA) method [50]. In the MM–PBSA method, the free energy of the protein–substrate binding, ∆*G*_bind_, is obtained from the difference between the free energies of protein–substrate complex (*G*_complex_) and the unbound receptor/protein (*G*_protein_) and ligand (*G*_ligand_) as follows

∆*G*_binding_ = ∆*G*_complex_ − [*∆G*_protein_ + ∆*G*_ligand_] (1)

where ∆*G*_complex_, ∆*G*_protein_, and ∆*G*_ligand_ are the free energies of the complex (protein and ligand). Each free energy term in Equation (1) was calculated with the absolute free energy of the species (protein, ligand, and their complex) in gas phase (*E*_gas_), the solvation free energy (*G*_solvation_), and the entropy term (TS) using (Equation (2)):

*G* = *E*_gas_ + *G*_solvation_ − *TS* (2)

*E*_gas_ is the sum of the internal strain energy (*E*_int_), van der Waals energy (*E*_vdW_), and electrostatic energy (*E*_ele_) (Equation (3)). *E*_int_ is the energy associated with vibrations of covalent bonds and bond angles, rotation of single bond torsional angles (Equation (4)):

*E*_gas_ = *E*_int_ + *E*_vdW_ + *E*_ele_ (3)

*E*_int_ = *E*_bond_ + *E*_angle_ + *E*_torsion_ (4)

The solvation free energy is divided into a polar part (∆*G*_PB_) and a nonpolar part (∆*G*_np_)

∆*G*_solv_ = ∆*G*_PB_ + ∆*G*_np_ (5)

Here, the polar contribution (∆*G*_PB_) is calculated by solving the Poisson–Boltzmann (PB) equation [54]. The value of the interior dielectric constant and exterior dielectric constant were set to 1 and 80, respectively. The nonpolar solvation energy (∆*G*_np_) was calculated from the solvent–accessible surface area (SASA) using the hard-sphere atomic mode. The probe radius of the solvent was set to 1.4 Å. ∆*G*_np_ is calculated using

Δ*G*_np_ = γΔSASA + β (6)

where the surface tension *g* and the offset β were set to the standard values of 0.00542 kcal·mol^−1^·Å^2^ and 0.92 kcal·mol^−1^, respectively.

Normal-mode analysis (NMA) is useful to estimate the change in solute entropy during ligand binding. However, the NMA calculation is considered to be problematic and time-consuming and the NMA approach also does not take into consideration the solvent entropy. In addition, the two substrates used in the present study are very similar. According to the previous studies [55,56], the entropy differences should be very small so that the correlation between the experimental *K*_m_ value and the calculated binding free energy may not be greatly improved. Therefore, the solute entropy term was neglected in the present study. For each MD-simulated complex, we calculated the ∆*G*_bind_ values for the 1000 snapshots of the MD trajectory (one snapshot for each 2 ps during the last 2000 ps of the stable trajectory) and the final ∆*G*_bind_ value was the average of the calculated ∆*G*_bind_ values for these snapshots.

## 4. Conclusions

Endo-1,4-β-xylanase (EC 3.2.1.8) is the enzyme from *R. albus 8* (Xyn10A) which catalyzes the degradation of arabinoxylan. In this study, we built the three-dimensional structure based on the known sequence of amino acids of this enzyme. Xyn10A binds the substrate with the −1 sugar in the ^2^*S_O_* conformation tighter than the substrate with the sugar in the ^4^*C*_1_ conformation. According to results obtained using MM-PBSA calculations, the most important subsite for the substrate binding is subsite −1. Computational alanine scanning of selected amino acid residues of the active site groove was carried out to elucidate the role of individual residues on the binding of the substrate with the −1 sugar in the ^2^*S_O_* conformations and the results indicate that Asn187 and Trp344 in subsite −1 may be important residues for substrate binding. The findings of the study presented here may be useful for further study on endoxylanases.
